# Translation of the Fear of Dental Pain Questionnaire in a Swedish Setting: Associations with Dental Anxiety

**DOI:** 10.3390/dj14050277

**Published:** 2026-05-07

**Authors:** Pontus Abrahamsson, Ulla Wide, Kajsa Nordberg, Magnus Hakeberg

**Affiliations:** 1 Department of Orofacial Pain and Jaw Function, Region Värmland, 65637 Karlstad, Sweden; pontus.abrahamsson@regionvarmland.se; 2 Institute of Odontology, Sahlgrenska Academy, University of Gothenburg, P.O. Box 450, 40530 Gothenburg, Sweden; kajsa.nordberg@vgregion.se (K.N.); magnus.hakeberg@odontologi.gu.se (M.H.)

**Keywords:** fear of dental pain, dental anxiety, questionnaire, psychometrics, adults

## Abstract

**Background/Objectives:** Fear of pain is common in both dental and health care settings and a factor that clinicians have to consider when examining and treating patients. Fear of dental pain has been described as associated with dental anxiety. The aims were to translate the Fear of Dental Pain Questionnaire—short form (s-FDPQ) into Swedish, to add three items, and to analyze the associations between fear of dental pain, dental anxiety and painful experiences of dental treatment. **Methods:** This cross-sectional study with a translation and validation component of the s-FDPQ, in a dental setting, included 178 adult individuals. Dental anxiety was measured with the Dental Anxiety Scale (DAS), and single questions captured experiences of dental care regarding pain. The analyses included exploratory factor analysis, internal consistency, intraclass correlation (ICC), and regression analysis (GLM). **Results:** Psychometric analyses revealed a two-factor solution, treatment and examination dimension, and the modified s-FDPQ (ms-FDPQ) had acceptable internal consistency = 0.87 and ICC = 0.93. Validation analyses using fearfulness of painful dental care and dental anxiety showed r = 0.75 and 0.68, respectively. In the GLM model, there were three significant predictors of fear of dental pain: women, dental anxiety and “fear of dental pain makes it more difficult to undergo dental treatment”. **Conclusions:** The ms-FDPQ shows acceptable validity and reliability as an instrument to measure fear of dental pain concerning several common procedures or treatments in routine dental care. However, the ms-FDPQ needs to be tested in other populations and settings. The results indicate that fear of dental pain is prevalent among adults.

## 1. Introduction

Fear of pain is common in both dental and health care settings and a factor that clinicians have to consider carefully on a daily basis when examining and treating patients. Fear of dental pain during treatment has been described as a central factor in the development of dental anxiety [1]. Dental anxiety is among the most commonly reported strong fears, with a prevalence of around 15% in the adult population, and approximately 5–10% have dental anxiety that is severe enough to be considered a specific phobia [2,3]. The etiology of dental anxiety is multifactorial, with individual, external and dental causal factors [4]. Key dental causal factors are earlier negative experiences of dental care, like painful dental treatments, and negative patient–dentist interactions. Individuals with severe dental anxiety report anxiety about various aspects of treatments, with pain generally being their primary concern [5].

In order to face challenges regarding fear of pain, reliable and valid questionnaires with appropriate psychometric properties are needed for both clinical and research purposes. One of the ways to assess fear of pain within dental care is the use of the Fear of Dental Pain Questionnaire (FDPQ). The FDPQ is based on the Fear of Pain Questionnaire (FPQ-III), which is a medical questionnaire developed by McNeil and Rainwater in 1998 [6]. The aim of the FPQ-III was to construct a screening instrument to analyze pain-related fear and allow identification of individuals in the healthcare system with extensive fear of pain. The questionnaire consists of 30 items related to possibly painful experiences, divided into three subscales—Severe pain, Minor pain, and Medical pain.

The original FPQ-III questionnaire includes a few items related to dental procedures. However, to make the instrument more suitable for dentistry, van Wijk and Hoogstraten developed a dental version of the questionnaire in Dutch, the Fear of Dental Pain Questionnaire (FDPQ). The questionnaire consists of 18 items describing dental procedures [7]. Later, a short version (s-FDPQ) was developed as a more efficient instrument in order to simplify the identification of patients with fear of dental pain. The short-version questionnaire consists of five items related to potentially painful dental procedures [8]. The items included are receiving an intraoral injection, drilling of a tooth, root canal treatment, extraction of a tooth (not wisdom tooth), and extraction of a wisdom tooth. The above five items are justified concerning possibly pain-inducing clinical procedures in the dental setting. However, other less invasive/hard tissue procedures can be relevant in measuring dental pain, such as probing teeth and periodontal pocket depth, as well as radiological intraoral examination.

The s-FDPQ has been translated from Dutch into several languages including English [8], Chinese [9], and Romanian [10]. Previous studies have shown promising results regarding reliability and validity for the s-FDPQ [8,9,10]. These findings indicate that the s-FDPQ is a useful tool to detect patients with fear of dental pain in clinical practice, and in research. There is no Swedish version of the s-FDPQ and thus there is a need for translation and psychometric evaluation of the questionnaire. The aims of this study were (1) to translate the s-FDPQ into Swedish, including modification of the number of items with three more items, and analyze the s-FDPQ with regard to psychometric properties, and (2) to analyze the association between fear of dental pain, dental anxiety and previous painful experiences of dental treatment in a clinical sample of adult individuals.

## 2. Materials and Methods

### 2.1. Design

This paper consists of a cross-sectional study with a translation and validation component of the s-FDPQ.

### 2.2. Translation and Modification of the s-FDPQ

The authors (K.N., P.A., M.H., and U.W.), who have clinical as well as psychological expertise in dental anxiety and phobia, translated the original s-FDPQ scale from English into Swedish according to Streiner’s guidelines [11]. Disagreements and different opinions regarding the translation were discussed among the authors during the process until consensus was reached within the group. A preliminary Swedish version of the s-FDPQ was constructed. The preliminary version was then translated back into English by a certified translator. A few differences were noticed between the original and the Swedish s-FDPQ, and these were rephrased and translated again to confirm the correctness of the translation. The preliminary questionnaire was tested on a small group of people in order to evaluate the comprehension of the Swedish translation. Feedback indicated that the questionnaire was overall easy to understand and only a few minor language changes were made before the final version was approved. Moreover, three items were added to include a broader variety of dental procedures with possible associations with fear of dental pain. The new items were “Having calculus removed”, “Having a dental examination with a dental probe”, and “Having an intraoral X-ray examination (sensor placed inside the mouth)”. The final version is called the ms-FDPQ (Fear of Dental Pain Questionnaire—modified short form).

### 2.3. Materials

A consecutive sample of individuals were recruited from five public dental service clinics in the region of Västra Götaland, Sweden. The inclusion criteria were age ≥ 18 years, understanding the Swedish language, and attending a general dental clinic. The participants were asked to answer a battery of questionnaires (in paper format) while in the waiting room, before their appointment with the dental professional. A power analysis was performed using alpha 0.05, power 0.80, effect size 0.50, and a difference of 2 s-FDPQ sum of scores, which resulted in a sample size of n = 129. We opted for a larger sample size to minimize the effect of non-participation. One hundred ninety individuals were invited to participate in the study, and n = 178 were included while n = 12 were excluded due to not meeting inclusion criteria or due to administrative errors. The Swedish Ethical Review Authority approved the study (Reg. No. 2021-00076). The study was conducted in accordance with the World Medical Association Declaration of Helsinki. Informed written consent was obtained from all participants.

### 2.4. Measurements

Background variables included gender (man, woman, other), age, level of education (elementary school, high school, university), dental care attendance (regular/every year or every second year; irregular/infrequently; only emergency visits/not at all) and self-rated oral health (bad, fair, good, excellent).

The Fear of Dental Pain Questionnaire—modified short form (ms-FDPQ) includes eight items related to fear of pain in the following dental procedures: “Receiving an anesthetic injection in the mouth”; “Having a tooth drilled”; “Receiving root canal treatment”; “Having a tooth extracted (other than wisdom tooth)”; “Having a wisdom tooth extracted”; “Having calculus removed”; “Having a dental examination with a dental probe”, and “Having an intraoral X-ray examination (sensor placed inside the mouth)”. The response was scored from 1 (no fear) to 5 (extreme fear). The total sum of scores varies from 8 to 40. For comparison purposes with previous studies, the sum of scores for the five-item s-FDPQ was also calculated, with a range of 5 to 25 [8].

Dental anxiety was measured using the Dental Anxiety Scale (DAS) [12]. The DAS consists of four items describing imagined dental situations including “appointment tomorrow”. Responses are scored from 1 (no anxiety reaction) to 5 (extreme anxiety reactions), giving total scores varying from 4 to 20 [13,14]. A DAS score ≥ 13 indicates high dental anxiety. The reliability has been shown to be reasonably high, with Cronbach’s α ranging between 0.62 and 0.91.

Experience of dental pain and fear of dental pain were measured with four single questions: “Are you fearful of painful dental treatment?”; “Does fear of dental pain make it more difficult to undergo dental treatment?” (response options: not at all, a little, moderately, a lot, extremely); “Did you experience pain during your last dental care visit?” (response options: no, yes a little, yes a lot); “Have you ever experienced pain during dental treatment?” (response options: no, one, several painful treatment(s)).

### 2.5. Statistical Analysis

The statistical analyses included measures of central tendency and distribution such as mean, median, standard deviation (SD), and range. Inference testing was performed using the *t*-test and the Mann–Whitney test and regression analysis using the General Linear Model (GLM). Reliability was evaluated using a test–retest, where a convenience sample (n = 36) of adult individuals completed the ms-FDPQ at two-week intervals. Reliability and validity were analyzed with intraclass correlation (ICC) and corrected item-total correlation and bivariate correlation analysis using the Pearson and Spearman methods. Moreover, internal consistency was analyzed with Cronbach’s alpha, while the dimensionality was tested with Exploratory Factor Analysis (EFA), including principal component analysis as the extraction method, while the rotation method applied was Varimax with Kaiser normalization. The level of statistical significance was set to *p* < 0.05. The GLM used ms-FDPQ as the continuous variable, and age, sex, education, the two single questions ‘Does fear of dental pain make it more difficult to undergo dental treatment?’ and “Experienced pain during previous dental visits”, and dental anxiety as independent variables.

## 3. Results

The sample consisted of 178 participants (64 men and 114 women) with varying levels of education and a mean age of 51.3 yrs (SD 17.5, range 18–90) (see Table 1). A majority of the participants reported regular dental visits (85%), and almost two thirds (65%) rated their oral health as good or excellent. The mean score of dental anxiety measured with Dental Anxiety Scale was 7.1 (SD = 3.2).

### 3.1. Reliability, Validity and Dimensionality of ms-FDPQ

The test–retest analysis showed good agreement, ICC = 0.93, 95% CI 0.87–0.97. For validity purposes, the ms-FDPQ sum of scores was correlated to the single question about fearfulness of painful dental care and DAS sum of scores, with a correlation coefficient of 0.75, and 0.68, respectively. Cronbach’s alpha for ms-FDPQ was 0.87, and for the s-FDPQ (5 item) 0.89. Further testing of ms-FDPQ with exploratory factor analysis together with corrected item-total correlations (Table 2) revealed that the X-ray examination item had the lowest correlation with the total sum of scores. However, Cronbach’s alpha did not vary substantially if any item was deleted (range 0.836–0.880). The EFA can be interpreted as having two dimensions. Factor 1 loads on invasive treatment procedures with loadings from 0.790 to 0.908, while Factor 2 loads on more examination procedures with loadings from 0.665 to 0.836. One item, anesthetic injection, has moderate loadings on both Factor 1 and Factor 2, 0.435 and 0.518, respectively. Using an Eigenvalue of one as the cutoff, Factor 1 and Factor 2 had 53.4% and 14.9% of explained variance, respectively, and thus a total of 68.3%.

### 3.2. Fear of Dental Pain Measured by the ms-FDPQ

The participants reported the highest level of fear of dental pain for “extraction of a wisdom tooth”, “root canal treatment”, “extraction of a tooth (other than wisdom tooth)”, and “drilling of a tooth” (see Table 3). The least fear of pain was reported for “X-ray examination” and “examination of teeth with a probe”. Statistically significant gender differences were found for all items as well as for the total sum of scores, with women scoring higher (except for the X-ray item). There were also statistically significant differences related to dental anxiety, where individuals with high dental anxiety reported higher levels of fear of pain on all items and a higher total score (see Table 3). These findings apply to both the ms-FDPQ (eight-item) and the s-FDPQ (five-item) total scores.

### 3.3. Single Questions Regarding Fear of Dental Pain and Experiences of Painful Dental Treatments

Most respondents had at some point experienced at least one painful treatment (74%) and the majority experienced a fear of having to go through painful treatments (78%) (see Table 4). Four out of ten respondents reported that the fear of pain makes dental visits more difficult. Women reported significantly higher levels of fear of painful treatments compared with men; however, there were no significant differences in any of the other questions between genders.

### 3.4. Multivariable Analyses

The multivariable GLM regression model (Table 5) revealed three significant predictor variables for the ms-FDPQ sum of scores with an adjusted R^2^ = 0.49. The variables gender, dental anxiety, and experiencing fear of dental pain making treatment difficult were significant predictors.

## 4. Discussion

This study reports on the adaptation of the s-FDPQ into a Swedish version, including the addition of three items. The results show acceptable psychometric properties for this modified short-form FDPQ (ms-FDPQ). A majority of the participants had experienced painful dental treatment and reported fear of dental pain. Furthermore, 40% stated that their fear of dental pain made dental visits difficult to cope with. Fear of dental pain was related to dental anxiety and gender.

The level of fear of dental pain among adults in the present study is in line with the results from previous studies using the s-FDPQ in the Netherlands and China [8,9]. However, a lower level has been reported in a Romanian study including only university students [10]. The Chinese and Dutch studies are based fully or in part on samples of individuals receiving dental care, like in the present study. Interestingly, 22% of the patients in the Chinese study had never seen a dentist when taking part in the study, compared to none in this study. For all the above studies, including the present, the ranking of the items with regard to the level of fear of pain shows a similar pattern [8,9,10].

The association between fear of dental pain and dental anxiety is obvious in the present study and is consistent with the results from the Chinese study [9]. This positive correlation between dental anxiety and fear of pain has also been confirmed in a study from Japan based on the FPQ-III [15].

While dental anxiety possibly decreases with age [5], Wright and McNeil found diverging results with respect to age and fear of pain [16]. That study did not use s-FDPQ, which makes relevant comparisons difficult with the present study, where the multivariable GLM revealed no statistical association between age and ms-FDPQ. Also, women reported higher fear of dental pain than men, which the GLM confirmed. This is in line with DiTella et al. [17], Roelofs et al. [18], and McNeil and Rainwater [6]’s findings on gender and fear of pain.

The ms-FDPQ was translated according to guidelines [11]. This strengthens the conceptual equivalence between the Swedish translation and the original questionnaire. The objective of modifying the questionnaire was to adapt it to a clinical setting and the most common procedures. Three items were therefore added to include examination of the teeth with a probe, having an intraoral X-ray and removing calculus, which were not included in the original s-FDPQ. This addition may further increase the applicability of the ms-FDPQ in both research and clinical settings and improve its discriminatory ability. The psychometric analyses show acceptable reliability outcomes, with both test–retest and internal consistency including high alpha values irrespective of whether items were deleted. Moreover, validity testing with ms-FDPQ and dental anxiety, and fearfulness of painful dental care indicate adequate levels. Interestingly, the EFA revealed two dimensions in the ms-FDPQ: factor 1, Treatment (4 items), and factor 2, Examination (3 items). However, the anesthetic injection item had moderate correlations on both dimensions (highest on factor 2). On the one hand, intraoral anesthetic injection may be perceived as an invasive procedure causing pain and can theoretically belong to Factor 1. On the other hand, intraoral anesthetic injection has the function of preventing pain from invasive procedures and can be interpreted to other dental procedures. Done et al. [10] performed a confirmatory factor analysis on s-FDPQ (including an additional item of dental implants) and found a one-factor solution. This finding is in line with the Treatment factor in the present study. The Examination dimension, with the three new items that clearly load on this dimension, may underscore the importance of including these three items in the ms-FDPQ. However, the psychometric properties should be further tested in other populations using both EFA and confirmatory factor analysis in order to understand its dimensionality.

This study has its limitations and strengths. The data were collected from individuals attending general dental public clinics, and the results may not be generalizable in a broader perspective, which may indicate selection bias problems when not using a random sample. Future studies with larger and more representative samples are needed to draw further conclusions for the general population. A further strength of this study is that the translation process followed Streiner’s guidelines for a structured translation process [11]. In addition, the ms-FDPQ was tested on a reasonably large clinical sample with varying levels of dental anxiety, similar to ordinary dental patients in Sweden [13], although the number of individuals with high dental anxiety was small. Moreover, the participants were included from several dental clinics from different regional areas, representing groups with varying social backgrounds.

## 5. Conclusions

The ms-FDPQ shows acceptable validity and reliability as an instrument to measure fear of dental pain concerning several common procedures or treatments in routine dental care. However, ms-FDPQ needs to be tested in other populations and settings, including further psychometric testing with EFA and confirmatory factor analysis, to interpret the dimensionality of the ms-FDPQ. The results indicate that fear of dental pain is common among adults, and that many adults have experienced painful dental treatment. Adequate pain relief and striving for pain-free treatment should be clinicians’ top priorities for all patients in order for the patient to cope with dental treatment.

## Figures and Tables

**Table 1:** Numbers (n) and proportions (%) by gender related to the background questions about education level, dental care attendance, experienced dental health and dental anxiety.

		Men n = 64 n (%)	Women n = 114 n (%)	Total n = 178 n (%)
Gender	Men	-	-	64 (36.0)
	Women	-	-	114 (64.0)
Education	Elementary school	12 (18.8)	14 (12.3)	26 (14.6)
	High school	33 (51.5)	54 (47.4)	87 (48.9)
	College/University	19 (29.7)	46 (40.3)	65 (36.5)
Dental care attendance	Regular/every or every second year	54 (84.4)	97 (85.1)	151 (84.8)
	Irregular	9 (14.0)	12 (10.5)	21 (11.8)
	Only emergency visits/ not at all	1 (1.6)	5 (4.4)	6 (3.4)
Oral health	Bad	7 (10.9)	5 (4.4)	12 (6.7)
	Fairly acceptable	13 (20.4)	37 (32.4)	50 (28.1)
	Good	37 (57.8)	57 (50.0)	94 (52.8)
	Excellent	7 (10.9)	15 (13.2)	22 (12.4)
Dental anxiety	No/moderate	61 (95.3)	104 (92.0)	165 (93.2)
	High	3 (4.7)	9 (8.0)	12 (6.8)

**Table 2:** ms-FDPQ items with corrected item correlations, explorative factor analyses (EFA) and Cronbach’s alpha.

Item	Corrected Item—Total Correlation	EFA Factor 1 Treatment	EFA Factor 2 Examination	Cronbach’s Alpha If Item Deleted
Anesthetic injection	0.550	0.435	0.518	0.865
Drilling of a tooth	0.809	0.796	0.390	0.836
Root canal treatment	0.721	0.829	0.241	0.846
Tooth extraction	0.738	0.887	0.176	0.844
Wisdom tooth extraction	0.742	0.908	0.153	0.843
Removal of calculus	0.512	0.229	0.743	0.868
Examination	0.592	0.238	0.836	0.861
X-ray examination	0.358	0.084	0.665	0.880

**Table 3:** Mean ($\overset{¯}{x}$) and standard deviation (SD) for the ms-FDPQ items and s-FDPQ items, in relation to gender and dental anxiety (DA) ^1^.

	Men n = 64 $\overset{¯}{x}$ (SD)		Women n = 114 $\overset{¯}{x}$ (SD)	No/moderate DA n = 165 $\overset{¯}{x}$ (SD)		High DA n = 12 $\overset{¯}{x}$ (SD)	Total n = 178 $\overset{¯}{x}$ (SD)
Anesthetic injection	2.1 (1.0)	**	2.6 (1.2)	2.3 (1.1)	**	3.8 (1.0)	2.4 (1.1)
Drilling of a tooth	2.5 (1.1)	**	3.0 (1.2)	2.7 (1.1)	**	4.5 (0.5)	2.9 (1.2)
Root canal treatment	2.8 (1.3)	**	3.4 (1.2)	3.1 (1.2)	**	4.7 (0.5)	3.2 (1.3)
Tooth extraction	2.9 (1.1)	*	3.3 (1.2)	3.0 (1.1)	**	4.6 (0.7)	3.1 (1.2)
Wisdom tooth extraction	3.0 (1.1)	*	3.4 (1.2)	3.2 (1.2)	**	4.7 (0.5)	3.3 (1.2)
Removal of calculus	1.7 (0.9)	*	2.0 (1.1)	1.8 (1.0)	**	2.8 (1.1)	1.9 (1.0)
Examination	1.4 (0.7)	*	1.7 (0.9)	1.5 (0.8)	**	2.6 (1.4)	1.6 (0.9)
X-ray examination	1.3 (0.7)		1.6 (0.9)	1.4 (0.8)	**	1.8 (1.1)	1.5 (0.9)
ms-FDPQ total	17.7 (5.9)	**	21.3 (6.3)	19.1 (5.9)	**	29.3 (4.4)	19.9 (6.4)
s-FDPQ total (5 items)	13.3 (4.8)	**	15.9 (4.7)	14.3 (4.6)	**	22.3 (2.3)	14.9 (4.9)

ms-FDPQ, Fear of Dental Pain—modified short form; s-FDPQ, Fear of Dental Pain—short form. * *p* < 0.05; ** *p* < 0.01. ^1^ One form missing due to an administrative error.

**Table 4:** Numbers (n) and proportions (%) by gender and total sample regarding single questions.

		Men n = 64 n (%)	Women n = 114 n (%)	Total n = 178 n (%)
Fear of painful dental experiences ^1^	Not at all	19 (29.7)	15 (13.2)	34 (19.1)
	A little	23 (35.9)	41 (36.0)	64 (36.0)
	Moderate	18 (28.1)	42 (36.8)	60 (33.7)
	A lot	4 (6.3)	11 (9.6)	15 (8.4)
	Extreme	0 (0)	5 (4.4)	5 (2.8)
Fear of dental pain makes dental treatment difficult ^2^	Not at all	41 (66.1)	65 (57.5)	106 (60.5)
	A little	13 (21.0)	29 (25.7)	42 (24.0)
	Moderate	4 (6.5)	11 (9.7)	15 (8.6)
	A lot	3 (4.8)	5 (4.4)	8 (4.6)
	Extreme	1 (1.6)	3 (2.7)	4 (2.3)
Experienced pain during last dental visit	No, no pain	37 (57.8)	72 (63.1)	109 (61.2)
	Yes, a little	26 (40.6)	40 (35.1)	66 (37.1)
	Yes, a lot	1 (1.6)	2 (1.8)	3 (1.7)
Experienced pain during previous dental visits	No painful treatment	20 (31.2)	26 (22.8)	46 (25.9)
	One painful treatment	16 (25.0)	41 (36.0)	57 (32.0)
	Several painful treatments	28 (43.8)	47 (41.2)	75 (42.1)

^1^ Significant difference between men and women, *p* < 0.05. ^2^ Two men and one woman had missing answers on this variable.

**Table 5:** General Linear Model ^1^ with the ms-FDPQ as the dependent variable and age, sex, education, previous dental care pain experiences, fear of pain and difficulty in receiving dental care, and level of dental anxiety as independent variables.

Independent Variables	B	95% CI	*p* Value
Age	−0.001	−0.046–0.043	0.959
Sex (ref. male)			
-women	2.013	0.536–3.490	0.008
Education (ref. university)			
-secondary	−0.423	−1.978–1.133	0.592
-elementary	−0.866	−3.249–1.518	0.474
Previous painful experiences (ref. no painful experience)			
-one or more painful experiences	0.486	−1.247–2.218	0.581
Fear of pain makes dental care more difficult (ref. not difficult)			
-little to extreme difficult	1.955	0.118–3.791	0.037
Dental anxiety	1.126	0.820–1.432	<0.001

^1^ Adjusted R^2^ = 0.49.

## Data Availability

Data is available upon reasonable request.

## Abbreviations

The following abbreviations are used in this manuscript:

FDPQ	Fear of Dental Pain Questionnaire
FPQ-III	Fear of Pain Questionnaire
s-FDPQ	Fear of Dental Pain Questionnaire—short form
ms-FDPQ	Fear of Dental Pain Questionnaire—modified short form
DAS	Dental Anxiety Scale
ICC	Intraclass Correlation
GLM	General Linear Model
EFA	Exploratory Factor Analysis
